# Institutional delivery services utilization and associated factors among mothers who gave birth in the last year in Mandura district, Northwest Ethiopia

**DOI:** 10.1371/journal.pone.0243466

**Published:** 2020-12-16

**Authors:** Kassahun Asres Mitikie, Gizachew Tadesse Wassie, Melkamu Bedemo Beyene

**Affiliations:** 1 Mandura District Health Office, Metekel Zone, Benishangul Gumuz, Ethiopia; 2 Department of Epidemiology and Biostatistics, School of Public Health, College of Medicine and Health Science, Bahir Dar University, Bahir Dar, Ethiopia; 1. IRCCS Neuromed 2. Doctors with Africa CUAMM, ITALY

## Abstract

**Background:**

The risk of death from complications relating to pregnancy and childbirth for women’s lifetime is higher in developing countries. Improving maternal and child health through a well-organized institutional delivery service is central to achieving reduced maternal and child mortality. Despite the efforts that have been made to improve maternal health outcomes in Ethiopia, institutional delivery is still unacceptably low.

**Objective:**

This study was conducted to assess institutional delivery service utilization and associated factors in the study area.

**Methods:**

A Community-based cross-sectional study was conducted. A multi-stage sampling technique was used to employ a total of 546 women. Data were collected using an interviewer-administered questionnaire and entered into EpiData version 3.1 and then exported to SPSS version 23.0. for analysis. Logistic regression models were used to determine factors associated with the outcome variable. Adjusted Odds ratios with 95% CI were computed to measure the strength of association and statistical significance was declared at p-value <0.05.

**Results:**

The Prevalence of institutional delivery in the study area was 38% (34%-42%). Factors significantly associated with institutional delivery were ANC visit 1.80 (1.12–2.91), knowledge of danger sign during pregnancy 3.60 (2.25–5.76), urban residency 2.09 (1.15–3.81), Parity 0.49 (0.25–0.95) accessibility of health facility 4.60 (2.01–10.89), husbands educational level: primary 2.50 (1.27–4.91), secondary and above 2.36 (1.24–4.48), mothers occupation: governmental employee 2.05 (1.00–4.18), and Private employee 2.42 (1.09–5.35).

**Conclusions:**

The prevalence of institutional delivery in the District was low. Antenatal visits, residency, knowledge of pregnancy danger signs, parity, and accessibility of health facilities, maternal occupation, and husband education were factors significantly associated with institutional delivery.

## Background

Institutional delivery is a delivery that has taken place in any medical facility owned by skilled delivery assistance [1]. Health care that a mother receives during pregnancy, at the time of delivery and soon after delivery is important for the survival and well-being of both the mother and the child. More than 20 million women worldwide become pregnant annually [2]. More than 71 percent of births were assisted by skilled health personnel globally in 2014, an increase from 59% in 1990 [3].

Globally, more than 70 percent of maternal deaths are due to five major complications (which are direct obstetric complications), hemorrhage (25%), infection (15%), the complication of unsafe abortion (13%), hypertension (12%) and obstructed labor (8%) [4]. Infections are the underlying causes in 11% of maternal, and one-fourth of newborn deaths, but the true burden of maternal infections and related complications remains unknown [5]. Over the past two decades, Latin America and the Caribbean region have made significant progress in reducing maternal morbidity and mortality that as a result of encouraging skill delivery. But maternal and child mortality and morbidity occurring in Asia and Africa have been leading the world in pregnancy-related complications [6].

As of 2015, the maternal mortality rate remains highest in sub-Saharan Africa at 546 maternal deaths per 100,000 live births compared to the global maternal mortality ratio(MMR) of 216 maternal deaths per 100,000 live births [7]. Most maternal deaths in Africa are related to direct obstetric complications that occur around the time of childbirth mainly hemorrhage, hypertension, sepsis, and obstructed labor, which combined account for 64% of all maternal deaths. The urban/rural divide also affects maternal, newborn, and child health and access to health care [8]. Mortality is consistently lower in urban areas than in rural areas with remote communities often having poorer access to health care [9]. An access to the skilled attendant at birth during antenatal care and delivery is promoted as a key strategy for improving maternal and newborn care in low and middle–income countries [10].

Despite the efforts that have been made in recent years to improve maternal health outcomes in Ethiopia, the proportion of women who receive assistance from skilled birth attendants are still unacceptably low [11]. Major causes of maternal deaths in Ethiopia are preventable that include antepartum and postpartum hemorrhage (APH and PPH), prolonged/obstructed labor and ruptured uterus, severe pre-eclampsia and eclampsia, sepsis, and complications of abortion which account for 69% of the deaths [12]. Ethiopia has been making significant progress on reducing maternal mortality and had achieved its millennium development goals(MDG) (to reduce maternal mortality) goal of 350 maternal deaths per 100 000 even though now stands at 353 in every 100,000 according to the 2013 United Nation(UN) and Ethiopian ministry of health estimate 2019 [13].

In Ethiopia, the percentage of live births delivered by a skilled provider remained virtually unchanged for a period of 5 years after 2000, but increased substantially after 2005; from 6% in the 2000 and 2005 Ethiopia Demographic Health Survey(EDHS) to 10% in 2011 EDHS, 26% in 2016 EDHS [14] and reached 50% in 2019 [15].

The maternal mortality rate declined from the 2011 EDHS estimate of 676 deaths per 100,000 to 412 deaths per 100,000 of 2016 EDHS. This remarkable decline in maternal mortality is due to slight increments in facility delivery [14]. The progress made so far in improving skilled delivery attendance has been impressive and the engagement of the Health Development Army (HDA) has been the engine for the achievement as compared to 26% of 2016 [19] which was 50% in 2019 EDHS [15].

Some previous studies from different parts of the world identified factors that lead to low utilization of health facilities for delivery service. These are related to the place of residence and socioeconomic statuses; such as women’s age, ethnicity, education, religion, culture, the clinical need for care, and decision-making power [1, 4, 12]. Even though, the maternal waiting room, ambulance service, women development army, and pregnant women panel discussion programs were installed to encourage institutional delivery service, the coverage of institutional delivery remains low.

Although many published and unpublished studies have been conducted regarding the utilization of institutional delivery services at the global and regional levels, the study area is uncovered yet. Therefore, this study was intended to assess the proportion of institutional delivery and to identify possible associated factors with it, in the context of the Mandura District.

## Materials and methods

### Study area

This study was conducted in the Madura district, which is found in the Metekel zone, Benishangul Gumuz regional state, North-west Ethiopia. The district is designated at 546 km from Addis Ababa and 338 km from the regional town, Assosa. The total population of district Madura projected from 2018 national census projected report was 56,760 for the year 2019. The pyramidal age structure of the population has remained predominately young with 47.4% under the age of 15 years of these children under five years of age accounts 16.18%. The average household size of the district was 4.5. Among the total inhabitants, 87% were Gumuze, Agew (8.9%), Amhara (3.9%), and all other ethnic groups constitute 0.2% of the population.

### Study design and period

Community based cross-sectional study was conducted from October 01 to October 30, 2019.

### Study population

All mothers who gave birth in the last year before the time of data collection in Mandura district of selected *kebeles* were included.

## Operational definition and definition of terms

### Institutional delivery service utilization

Refers to mothers who had delivered their last baby in hospitals, health centers, private clinics, NGO health facilities, or Health Posts by skilled personnel [16].

### Skilled birth attendants

Refer to people with midwifery skills (midwives, doctors, and nurses with additional midwifery training) who have been trained to proficiency in the skills necessary to manage normal deliveries and diagnose, manage or refer obstetric complications’.

### Accessibility of institutional delivery service

Availability of heath facility providing delivery service within 2 hours distance by walk or <5 km [16].

### Predisposing factors

Are factors that exist prior and make susceptible or inclining to acquire some behavior like the use of skilled birth attendants [17].

### Enabling factors

Are usually thought of as barriers to behavior changes created by societal factors; such as availability of services and their accessibility (both geographic and economic), limited facilities, and lack of income.

### Reinforcing factors

Are the influences of people that encourage or discourage behavioral change [17].

### Antenatal care visitor

If a woman visited health care facility during pregnancy for getting pregnancy-related service.

### Access to health care facility

If a woman traveled <5 km to reach the nearest health care facility, considered as she has access to health care facility.

### Home delivery

When a mother gave birth at her home or others’ home (neighbor, relatives, or family) or when a birth takes place outside of health institution.

### Woman’s autonomy

If a woman decided on the place to give birth by herself or with her husband jointly, she would consider as autonomous.

### Women’s knowledge

A woman would be considered knowledgeable for danger signs of pregnancy if she scores 50% and above for knowledge questions categorized as have good knowledge otherwise poor knowledge when one is given for correct answer and zero for incorrect answer [16].

## Sample size determinations

The sample size was calculated using factors significantly associated with institutional delivery service utilization from previously conducted studies. It was calculated using STATA software by considering the assumptions of the 95% level of confidence, 5% margin of error, 1:1 case to control ratio, and 80% power of the study. The factors like place of residence, availability of radio/TV, and Educational status of the mother taken from a study conducted in Checha district, Pawe district with the same topic [18, 19] respectively. The maximum sample size was 248. Considering design effect of 2 and the potential non-response rate of 10%; the final sample size was 546.

## Sampling method and procedure

**Fig 1:**
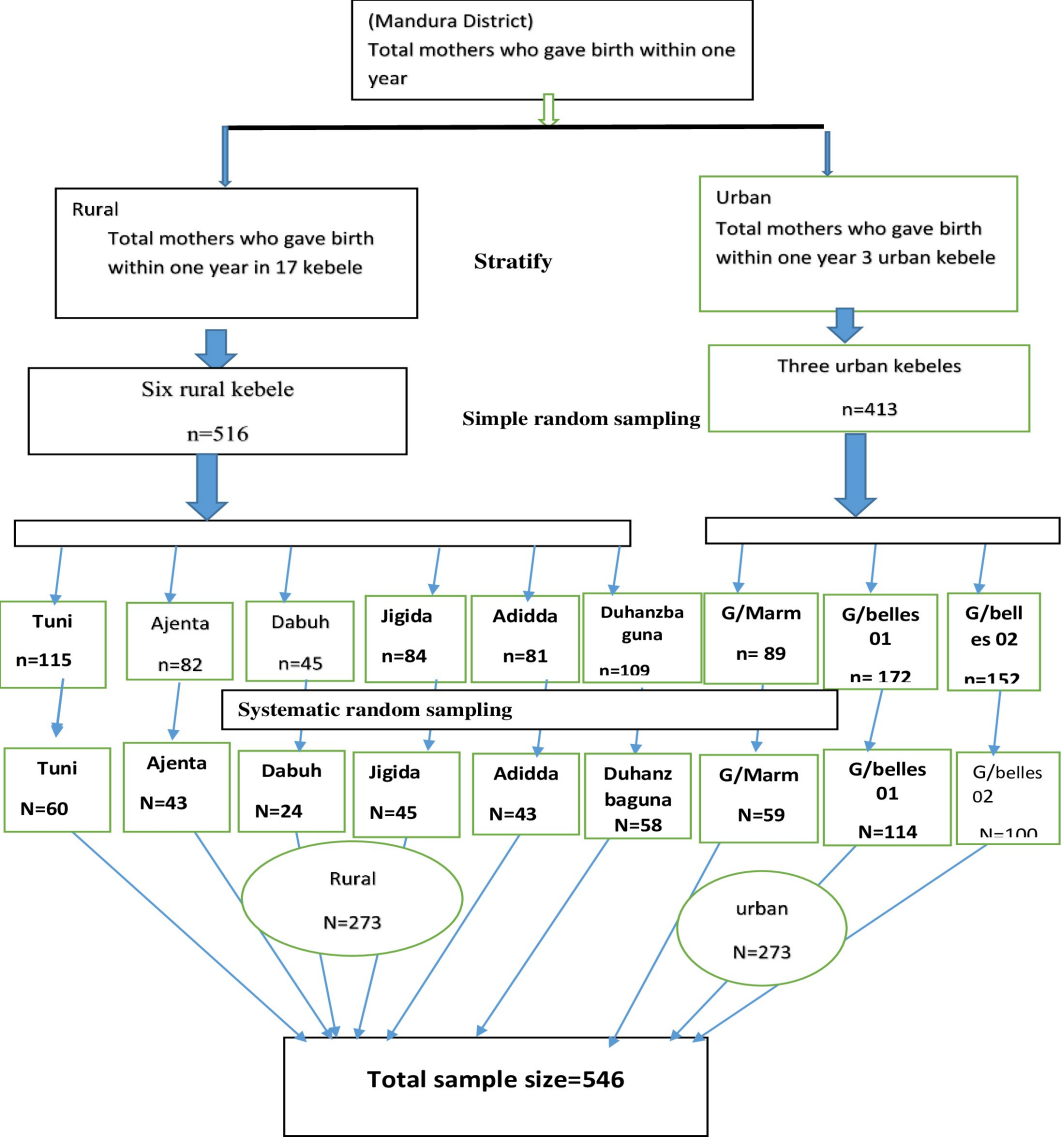
Schematic presentations of sampling procedures on delivery service utilizations and associated factors among mothers in Mandura district, 2019.

Multi-stage sampling was employed. First, stratification of the study area by urban and rural was done. Second, Random sampling was held from seventeen rural and all three urban kebeles (lowest administrative unit in Ethiopia) to select a total of nine kebeles; three from urban and six from rural. Then, Systematic random sampling was used with every K^th^ (two) cases from each kebele to get representative participants. Proportional sampling was done based on the number of mothers who gave birth in the last year living in the selected kebele using last year's pregnant mothers' registration book as sampling frame in the health post (Fig 1).

## Data collection instruments and procedures

Pre-tested questionnaires were used for data collection. The questionnaire was translated into the local language Amharic, and to check for its consistency, back-translated into English Data were collected by trained team through face to face interview. The questionnaire contains, questions developed from literatures done on delivery service utilization and questions developed by student investigators. The main domains of the questionnaire were predisposing factors, enabling factors, reinforcing, and need factor on potential associated factors for institutional delivery service utilization.

Nine health extension workers were recruited as data collector to conduct the face-to-face interview and two BSC degree nurses and the Principal investigator supervised the data collection process. Two days of training was given to the data collectors and supervisors before the actual data collection regarding the aim of the study, data collection tool, and procedures going through the questionnaires. The collected data were checked for completeness and consistency daily by supervisors. Before the actual data collection took place, a pre-test was done on 5% of the participant out of the study area to ensure the validity of data collection tools. Based on the findings of the pre-test, data collection tools were modified. After checking for completeness, the collected data were checked and reorganized to exclude errors before data entry.

### Statistical analysis

The collected data were checked for completeness and entered into EpiData version 3.1 software programs and then exported to SPSS version 23 software programs for data cleaning and analysis. Both descriptive and analytical analyses were carried out to describe the study subjects’ characteristics and to see the crude and adjusted effect of each variable. The binary logistic regression model was fitted to assess the effect of each independent variable towards the acquisition of institutional delivery service utilization. To identify factors associated with delivery service utilization among women in the Mandura district. First, bi-variate logistic regressions were performed for each independent variable with institutional delivery service utilization and crude odds ratio with 95% confidence intervals were obtained. Then, variables observed with P value<0.2 in the bi-variate analysis were subsequently included in the multivariate logistic regression to determine the independent predictors, and model fitness was checked by the Hosmer–Lemeshow goodness-of-fit and Potential multi-collinearity among the independent variables was also checked using variance inflation factor (VIF) at the maximum threshold of 10, and no multicollinearity was detected. The findings of the study were presented using text, tables, and graphs.

## Ethics approval and consent to participate

Ethical approval was taken from Bahir Dar University, Institutional review board of college of medicine and health sciences (protocol number: 020111/18.09). Official letters were written to the respective officials of the Mandura health office to obtain permission. After giving information and thoroughly explaining the aim of the study to the head office, permission was obtained. Written consent was taken from participants. Participants were assured that data would not be disclosed to anybody, they were not requested to give their name, and information was kept confidential as well as their privacy.

## Results

### Socio-demographic characteristics of the respondents in the district

A total of 546 mothers who gave birth in the last 12 months were interviewed, making a response rate of 100%. The mean age with a Standard deviation (SD) of the respondents was 27.8 (±5.9). The majority ethnic groups, 294 (53.4%) were Gumuz, whereas Amhara accounts 128 (23%). Orthodox Christianity was followed by 401 (73.4%) and Muslim religion practiced by 49 (8.9%). The majority, 528 (96.7%) of women were married.

Regarding educational status, 319 (58.4%) of mothers and 296 (54.2%) of their husbands were unable to read and write. Four hundred thirteen 413 (75.6%) of the mothers were housewives, while 348 (63.7%) of the husbands were farmers. Nearly half of respondents households 260 (47.6%) had an average monthly income of between 500–1500 Ethiopian Birr(ETB); whereas 123 (22.5%) respondents household had an average monthly income of between 1501–2500 ETB and the highest monthly income accounts 110 (20%) which was estimated to be greater than 2501 ETB.

Two hundred thirty (42.1%) respondents had either radio or TV. Two hundred seventy (49.4%) respondents had to walk for less than 30 minutes on average while 140 (50.7%) rural participants had to walk for more than 30 minutes on average to reach the nearest health facility. About, 413 (75.5%) of participants were housewife followed by 15.2% governmental employees. Three hundred forty eight (63%) husbands were farmers and 114 (20.9%) were governmental employees (Table 1).

**Table 1:** Socio-demographic characteristics of the study participants in Mandura district, Northwest Ethiopia, 2019 (n = 546).

Variables	Categories	Frequency	(%)
Age	<19	40	7.33
	20–34	434	79.5
	>34	72	13.2
Marital status	Married	528	96.7
	Divorced/widowed/	18	3.3
Religion	Orthodox	401	73.4
	Muslim	49	9.0
	Protestant	37	6.8
	Catholic	30	5.5
	Other***	29	5.3
Ethnicity	Amhara	128	23.4
	Oromo	31	5.7
	Agew	69	12.6
	Gumuze	294	53.8
	Shenasha	24	4.4
Mothers occupation	House wife	413	75.6
	Government employee	83	15.2
	Others	50	9.2
Mothers education	Unable to read and write	319	58.4
	Primary (1–8)	100	18.3
	Secondary and above	127	23.3
Income	<500	53	9.7
	500–1500	260	47.6
	1501–2500	123	22.5
	>2501	110	20.1
Husband’s education	Unable to read and write	296	54.2
	Primary education (1–8)	83	15.2
	Secondary and above	167	30.6
Husband’s occupation	Farmer	348	63.7
	Government employee	114	20.9
	Other**	84	15.4
Means of communication	Radio	36	6.6
	TV	194	35.5
	None	316	57.9

## Obstetric characteristics of the respondents in Mandura district

One hundred sixteen (39.4%) of mothers were given their 1^st^ birth before the age of eighteen. Three hundred three (55.5%) of respondents were reported that their last pregnancy was not planned. Majority, 472 (86.4%) of mothers had ANC visits in which 166 (30%) of them had four visits, 168 (30.8%) three visits, 119 (21.8%) two visits, followed by 36 (6.6%) one visit.

About, 438 (80.2%) of the respondents had an access to health facility service within a 5 km radius. Only, 222 (40.7%) of mothers had good knowledge about the danger signs of pregnancy and delivery-related complications. Disproportionate number of rural and urban residents, 245 (89.7%) of rural and 40.6 (40.6%) of urban residents had no transportation access to visit health care facilities. About, 90.7% of respondents decided their place of delivery by themselves and 343 (62.8%) of mothers knew their husband would prefer to deliver at health institutions. However, 26 (9%) of them did know about the preference of their husband for the place of delivery (Table 2).

**Table 2:** Obstetric related characteristics of respondents in Mandura district, Northwest Ethiopia, 2019 (n = 546).

Variables	Categories	Number	Frequency %
Walking time taken to the nearest health facility	< = 30 mint	270	49.5
	>30 mint	276	50.5
Age of the mother at 1st birth	< = 18	215	39.4
	>18	331	60.6
Number of parity	1	130	23.8
	2–4	298	54.6
	= >5	118	21.6
Number of live birth	1	135	24.7
	2–4	293	53.7
	> = 5	118	21.6
Types of pregnancy	Planned	303	55.5
	Not planned	243	44.5
Have ANC visit	Yes	472	86.4
	No	74	13.6
Number of ANC visits	No visit	57	10.4
	1 visit	36	6.6
	2 visits	119	21.8
	3 Visits	168	30.8
	4 visits	166	30.4
Place of ANC follow up	Health post	277	50.7
	Health center/Hospital	218	39.9
	Home	7	1.3
Knowledge status	Good knowledge	222	40.7
	Poor knowledge	324	59.3
Health facility with delivery service in the kebele<5km	Yes	438	80.2
	No	108	19.4
Availability of transportation to visit the health facility	Yes	190	34.7
	No	356	65.2
Cost affordability to pay for transportation	Yes	199	36.4
	No	335	61.4
Decision-maker place of delivery	Myself	495	90.7
	Family members	49	9.0
Preference of husband for the place of delivery	Home delivery	56	10.3
	Institutional delivery	343	62.8
	Do not know	147	26.9
Preference of husband as an attendant	Skilled delivery attendant	306	56.0
	Family members	237	43.4

## Institutional delivery service utilization in Mandura district

**Fig 2:**
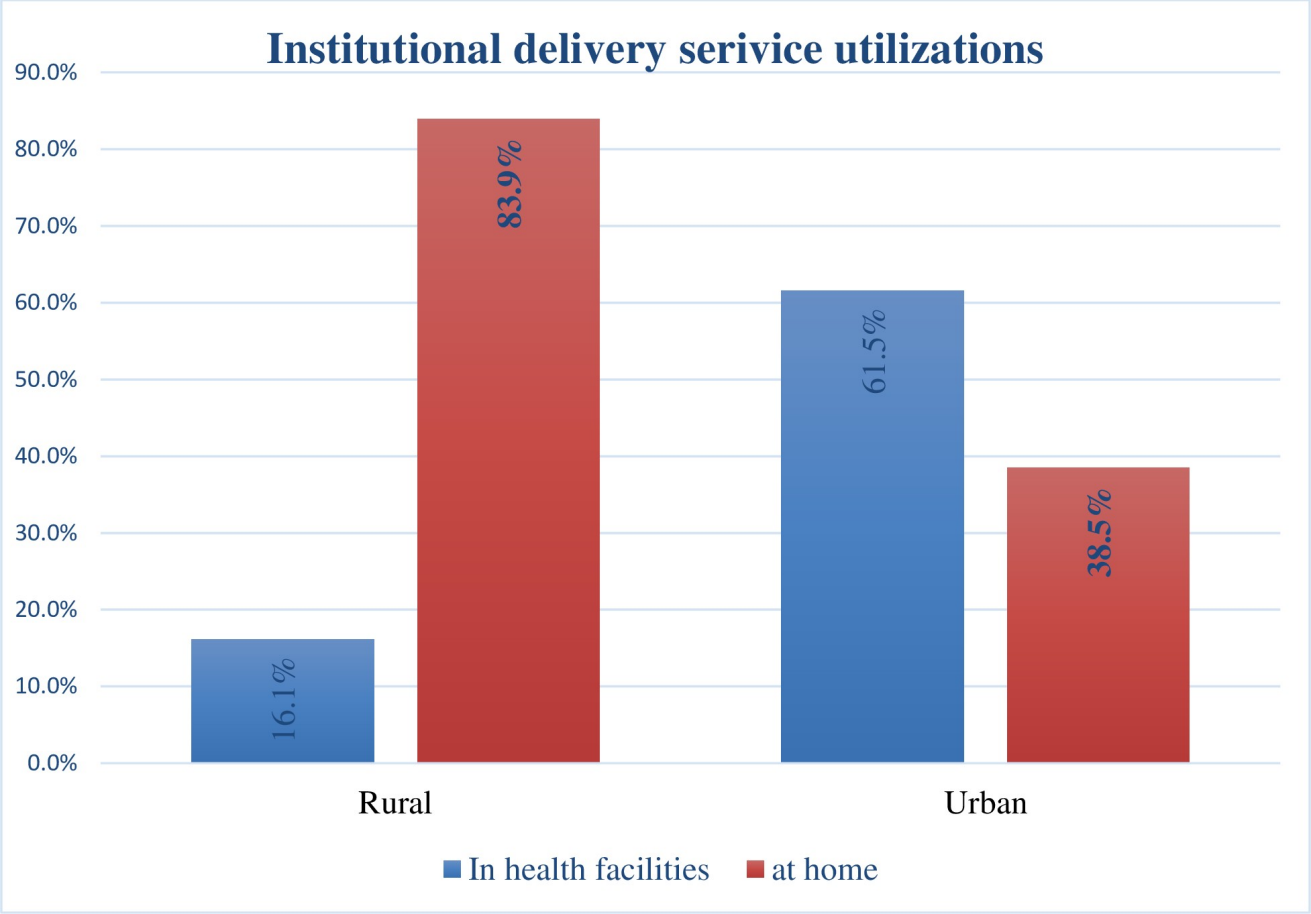
The proportion of women utilizing institutional delivery service among urban and rural in Mandura district, 2019.

The prevalence of institutional delivery service utilization in the Mandura district was 38.8% with 95% CI (34%-42%). Women from the urban area were more likely to receive delivery care from health facility than women from a rural area (Fig 2).

**Fig 3:**
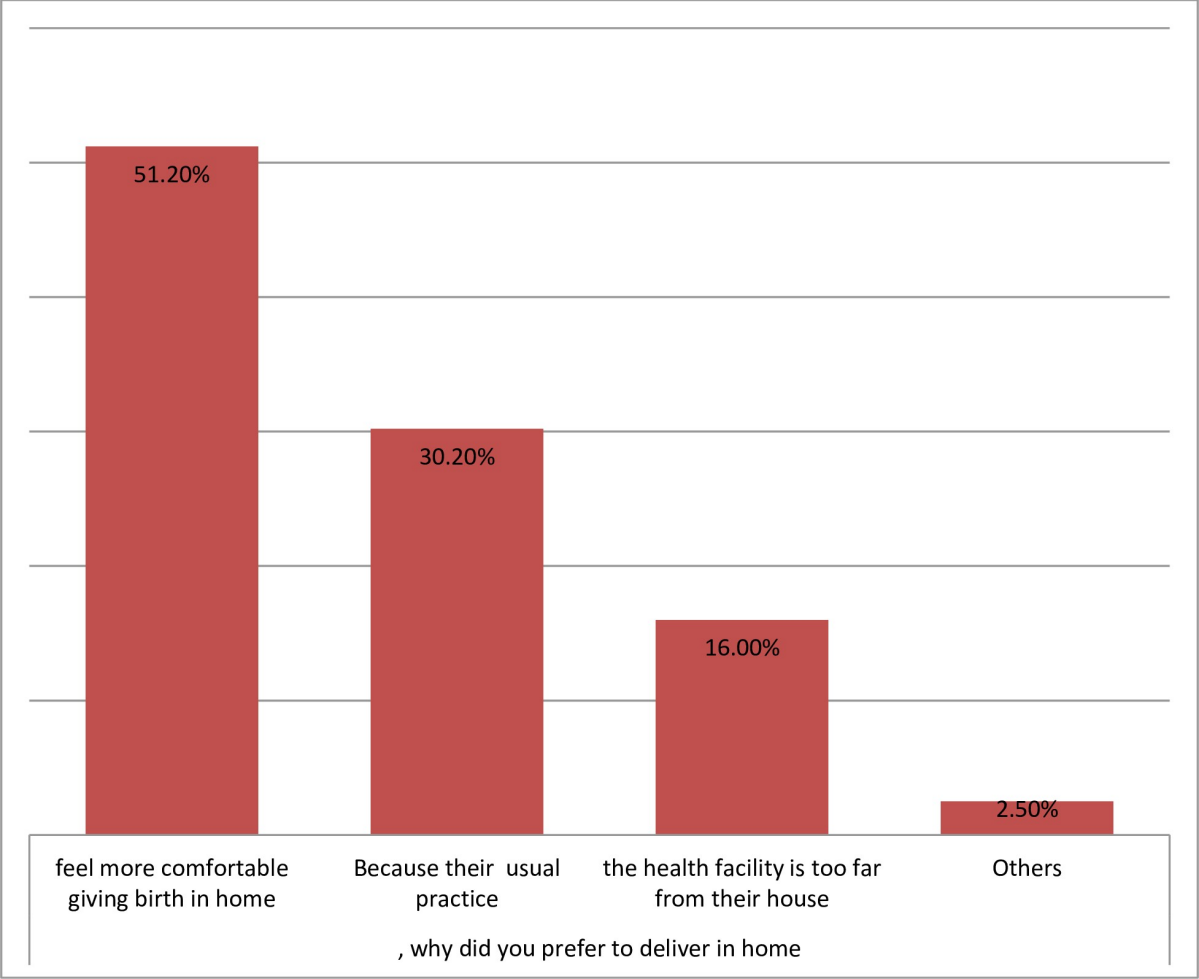
Reasons of women gave birth in home among study subjects in Mandura district, 2019.

**Fig 4:**
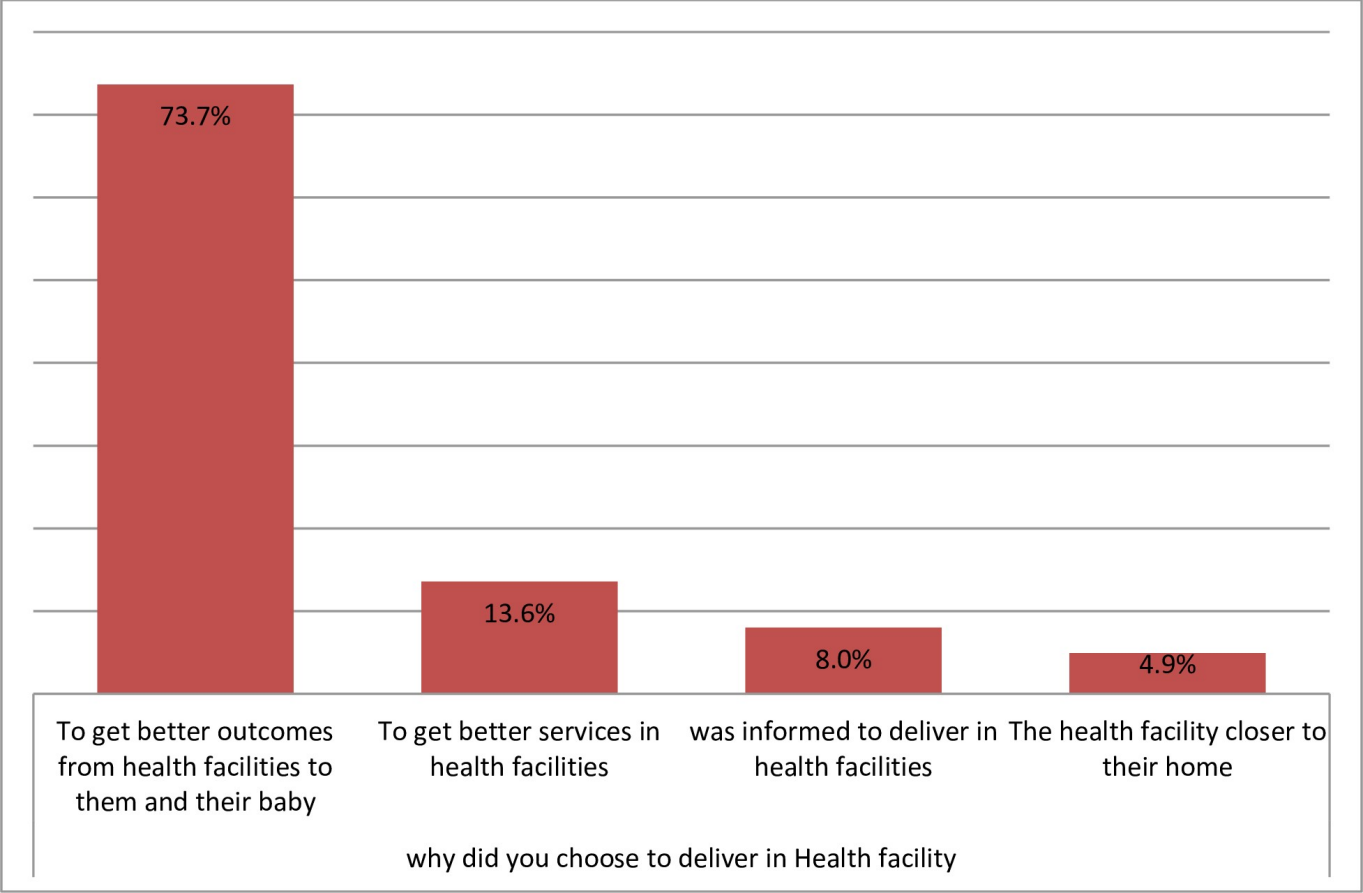
Reasons of women utilized institutional delivery service among study subjects in Mandura district, 2019.

Mothers gave a variety of reasons for preferring to deliver at home. Of these, feel more comfortable (51.2%), it is their usual experience accounts (30.2%) and the health facilities were too far from their house (16%) were among the commonest reasons (Fig 3). The reasons given to deliver at health institutions presented under (Fig 4).

## Factors associated with institutional delivery service

Bi-varaite analysis gave; place of residence, mothers education, husbands education, mothers occupation, husbands occupation, accessibility of health facility, health facility distance, means of communication, availability of transportation, frequency of ANC visit, parity and knowledge of the mother on pregnancy-related complications were candidate variables for multi-varaite analysis with a p-value of <0.2. The multi-varaite analysis revealed that residence of women, women occupation, husband education, frequency of ANC visit, accessibility of health facility with delivery within 5 km, parity, availability of transportation, and knowledge had a significant association with using institutional delivery.

Women from urban areas were 2.09 times more likely to use institutional delivery service as compared to rural women (AOR = 2.09, 95%CI:1.15–3.81). Government employee women were 2.05 times more likely to utilize institutional delivery as compared with housewife women(AOR = 2.05, 95%CI: 1.00–4.18) and private/self-employed women were 2.42 times more likely to utilize institutional delivery service as compare to housewife (AOR = 2.42, 95%CI: 1.09–5.35). Women, whose husband with primary educational status were 2.50 times more likely to utilize institutional delivery service than mothers who had husbands unable to read and write (AOR = 2.50, 95%CI:1.27–4.91). Similarly, women whose husband's educational status was secondary and above were 2.36 times more likely to utilize institution delivery service than mothers who had husbands unable to read and write (AOR = 2.36, 95%CI:1.24–4.48) (Table 3).

**Table 3:** Factors associated with institutional delivery service in Mandura district, Northwest, Ethiopia, 2019 (n = 546).

Variable	Category	Place of delivery		COR (95%CI)	AOR (95%CI)
		Health facility	Home		
Residence	Rural	44	229	1	1
	Urban	168	105	8.32 (5.5–12.4)**	2.09 (1.2–3.8)**
Occupation	Housewife	112	30	1	1
	Governmental employee	63	20	8.5 (4.89–14.)*	2.05 (1.00–4.18)*
	Private employee /merchant	37	13	7.6 (3.9–14.9)*	2.4 (1.1–5.4)*
Educational status of the mother	Unable to read and write	69	250	1	1
	Primary education (1–8)	51	49	3.8 (2.3–6.1)*	0.7 (0.3–1.7)
	Secondary and above	92	35	9.5 (5.9–15.3)*	1.02 (0.39–2.7)
Average monthly household income	<500	16	37	1	1
	500–1500	69	191	0.8 (0.4–1.5)	0.5 (0.2–1.2)
	≥1501	127	106	1.6 (0.8–3.2)	0.4 (0.1–1.2)
Husband’s education	Unable to read and write	57	239	1	1
	Primary (1–8)	37	46	3.4 (2.0–5.7)*	2.5 (1.3–4.9)*
	Secondary and above	118	49	10.1 (7–15.7)	2.4 (1.2–4.5)*
Husband’s occupation	Farmer	75	273	1	1
	Gove employee	83	31	9.7 (5.9–15.8)*	2.5 (0.8–7.0)
	Others***	54	30	6.55 (3.9–10.95)*	2.6 (.95–6.4)
Means of communication	Radio/TV	135	95	1	1
	None	77	239	.23 (0.16–0.3)	0.9 (0.47–1.89)

*P. Value <0.05

**Value<0.01

*** (Others)-merchant, private employer, daily labor.

Women who had at least three and above ANC visits during their last pregnancy were 1.80 times more likely to utilize health facility delivery service than attending at least two or less (AOR = 1.80, 95%CI:1.12–2.91). Women who had good knowledge about the danger signs of pregnancy and delivery related health problems were 3.60 times more likely to utilize institutional delivery service than women who had poor knowledge (AOR = 3.60, 95%CI:2.25–5.76).

The number of parity also had a statistical significant association with institutional delivery. Women who had two and more parity were 49% less likely to deliver at the health care facility as compared to Para one (AOR = 0.49, 95%CI: 0.25–0.95). Women who had an access to a health facility with delivery service were 4.6 more likely to utilize institutional delivery service than women who had no access to delivery services within a 5 km radius (AOR = 4.6, 95%CI: 2.01–10.89) (Table 4).

**Table 4:** Factors associated with institutional delivery service utilization among mothers who gave birth in the last year in Mandura district, Northwest, Ethiopia, 2019 (n = 546).

Variable	Categories	Place of delivery		COR (95%CI)	AOR (95% CI)
		Health facility	Home		
Time to the nearest health facility	< = 30	94	159	1	1
	>30	118	175	0.2 (.13–0.29)*	1.42 (0.82–2.45)
Age at 1^st^ birth	< = 18	69	146	1	1
	>18	143	188	1.60 (1.12–2.30)*	0.59 (0.33–1.06)
Parity	1	75	55	1	1
	2–4	101	197	0.37 (0.24-.574)*	0.49 (0.25–0.95)*
	= >5	36	82	0.32 (0.19–0.54)*	0.82 (0.34–1.96)
Knowledge status	Poor knowledge	50	231	1	1
	Good knowledge	162	103	7.26 (4.9–10.7)**	3.6 (2.25–5.76)**
Health facility accessibility	No	9	99	1	1
	Yes	203	235	9.5 (4.6–19.2)	4.46 (1.9–10.2)*
Decision maker for the place of delivery	Herself	185	310	1	1
	Family members***	27	22	2.05 (1.13–3.71)*	2.5 (0.73–8.8)
Number of ANC visits	< = 2 visit	64	148	1	1
	> = 3vist	148	186	1.84 (1.27–2.64)*	1.80 (1.12–2.9)**

*P. Value <0.05

**p-value<0.01

*** Family members (husbands, mother-in-law relatives).

## Discussion

Institutional delivery service is the most proven intervention in reducing maternal and child Mortality and disability. This study revealed that, the proportion of women who gave birth in a health institution in the district was 38.8% with 95% CI (34%-42%). This result was consistent with that of a study done in Assayita district, North East Ethiopia which was 36.1% [20]. However, it is higher than studies conducted in the Assosa zone, which was (24.8%) [1] and Checha district, Gurage zone, Ethiopia which was 31% [18]. This improvement might attributed by multipurpose health extension workers and women development army who have been playing a pivotal role in providing information on maternal and child health services. However, it was lower as compared to studies conducted in Pawe district, which was 60% [19], Arbaminch Town, Gamo Gofa Zone, southern Ethiopia which was 73.2% [21], EDHS 2019 which was 48% [15], Woldia Town, Ethiopia was 74.7% [22], and Mizan Aman City administration, South West Ethiopia was 54.2% [23].

Place of residence was another significantly associated factor with institutional delivery service utilization. Urban women were more likely to give birth at health institutions as compared to their rural counterparts. This finding was in line with previous studies conducted in Nigeria and other parts of Ethiopia; Ayssaita District, Raya, Alamata District, and Mizan-Aman Town [17, 20, 24, 25] respectively. This might be urban women would have an increased access to health facility delivery service, and transportation, and information than rural women. In addition, ANC utilization which has a direct relationship with institutional delivery utilization is usually better utilized in urban settings than rural [14].

Women who had good knowledge of pregnancy and delivery related problems were more likely to be utilized institutional delivery service than those who had poor knowledge. This result was in line with previous similar studies conducted in developing countries [19, 22, 24, 26, 27] which revealed that delays in seeking health care during pregnancy are influenced by individual and community knowledge on maternal health care services. Besides, knowledge is an important factor that affects attitude, practice, and health seeking behavior of the people. Therefore, women who had good knowledge about danger signs of pregnancy would speculate the potential adverse pregnancy outcomes; as a result they would be motivated to deliver at the health facilities.

Women who had access to health facilities were more likely to utilize institutional delivery service. This finding is in line with studies [18, 28, 29]. This could be explained by an increased distance from the health facility, transport cost, and lost production time, as well as possible lower exposure to health information.

Women who attended antenatal care visits at least three and more times were more likely to use institutional delivery care than women who attended two or less than two, and not attended. This finding was supported by different studies done in Nepal [23], Assosa district, Benishangul Gumuz Regional State, Western Ethiopia, Oromia Ethiopia, Ayssaita district, Northeast Ethiopia and Liben zone, Somalia regional state [1, 20, 30, 31] respectively. This might be because antenatal services can provide opportunities for women to obtain information on the status of their pregnancy, which in turn alerts them to decide where to deliver. It is also a fact that many ANC visits expose the women to more health education and counseling which could both likely to increase service utilization. Besides, the use of ANC might signify the availability of nearby health institutions provided delivery care service.

Parity was also a significant factor that was associated with the utilization of institutional delivery service. Women with lower parity were more likely to deliver in the health facility. This finding was in line with several other studies from Nepal [23] and in our country in different areas [17, 26]. This could be explained that women with lower parity tend to give careful attention to seeking delivery assistance due to their lack of experience in pregnancy and fear of complication. Conversely, women with more children believe themselves to be more experienced in childbirth, and for this reason; they might less likely to use facility delivery service.

Husband’s educational status was an important factor to allow women to utilize institutional delivery service. Women who had husbands with at least primary and above level of education were more likely to give birth in health institutions. This finding was consistent with studies done from pastoralist community, in Afar, Northeast Ethiopia [32], Meta-analysis in Ethiopia [28], in Bench Maji zone, South West Ethiopia [23]. The possible justification for this could be educated husbands might have a better understanding of the complication of home delivery and the benefit of institutional delivery. Therefore, they might assist their partner in deciding on the place of delivery that could be understood the increasing level of education in the community had a contribution to increasing institutional delivery service utilization.

Women’s occupational status was also significantly associated with institutional delivery services utilization. Government and private employee women were more utilized institutional delivery service as compare to housewives. The finding is consistent with other studies which were conducted in Dangila district [33], Afambo district, Afar, Ethiopia [16], Pastoralist Community of in Afar, Northeast Ethiopia [32]. Women’s employment could be attributed from a higher level of education with better knowledge and more autonomous with control of their earning in seeking care. However, housewives are less likely to know about pregnancy and childbirth due to lesser freedom of movement outside the household and less likely to seek information services importance for pregnancy care that might decrease institutional delivery serves utilizations.

## Limitations of the study

Since the women were asked for their exposure retrospectively, the response might be susceptible for recall bias. In addition, the cross-sectional nature of the study design does not allow demonstrating causation.

## Conclusions

This study revealed that the utilization of institutional delivery service in the study area was low with a high disparity of urban and rural residents as compared to the national level. Factors identified associated with institutional delivery service utilization were: the place of residence; husbands’ educational status, number of ANC visits, occupational status of women, parity, and women’s knowledge of danger signs during pregnancy, and accessibility of health facility with delivery service. Promoting the education of the women and their husbands, empowering women to have a job, proper utilization of ANC services, improving the infrastructure for accessibility of health care facilities, and ensuring women’s knowledge towards danger signs during pregnancy and the benefits of institutional delivery are the preliminary actions to be considered by the health care providers, district health office and local movement administers.

## Supporting information

S1 File (PDF)

S2 File (PDF)
