# Supplementary material for: Institutional delivery services utilization and associated factors among mothers who gave birth in the last year in Mandura district, Northwest Ethiopia
Source: PLoS One. 2020 Dec 16;15(12):e0243466. doi: 10.1371/journal.pone.0243466 (PMC7743934; doi:10.1371/journal.pone.0243466)
Supplement: S1 File — (PDF) [file pone.0243466.s001.pdf]

Annex I: participant information sheet and questioners (Amharic)

ID-------------------------------

ጤና ይስጥልኝ ስሜ------------------------------ይባላል፡፡ ይህ መጠይቅ የተዘጋጀበት ዓላማ በማንዱራ ወረዳ ባለፉት አስራ ሁለት ወራት የእናቶች በጤና ተቋማት የመዉለድ ሁኔታወች እና የእናቶች በጤናተቋማት እነዲወልዱ የሚያደርጉ ሁኔታወች በተመለከተ መረጃ ለመሰበሰብ እና ለማጥናት ነዉ፡፡እርስዎ እዚህ ጥናት ዉስጥ እንዲካተቱልኝ ሰለመረጥኩዎት ለምጠይቀዎት ጥያቄዎች መረጃዎች በመስጠት እንዲተባበሩኝ በትህትና እጠይቀዎታለሁ፡፡

በዚህ መጠይቅ ዉስጥ የሚካተቱ ጥያቄዎች ሰለማህበራዊ እና ሰነ-ህዝበ ሁኔታ፣ስለባለፈዉ የፅንስ እና የወሊድ ሁኔታ፣ስለባለፈዉ የእርግዝና ክትትል እና የወለድሽበት ቦታ፣እንዲሁም ስለ በቤት ዉስጥ ዉሳኔ ስለመስጠት የመሳሰሉት እና ሌሎችም ተካተውበታል፡፡ሁሉም የምትሰጭኝ መረጃወች በሚስጥር ይያዛሉ፡፡በዚህ መጠየቅ ዉስጥ የአንቺ መለያዎች የሚሆኑ ማለትም ስምሽና ትክክለኛ አድራሻሽ ኣይካተቱም፡፡የምትሰጪቸዉ ትክክለኛ መልሶች ብቻ ናቸዉ የወረዳዉን የእነቶች በጤና ተቋማት የመዉለድ ሁኔታወች አንድናዉቅ የሚረዱን፡፡የጥናቱ ተሳታፊ በመሆንዎ በቀጥታ እርሰዎን ተጠቃሚ ሊያደርግ አይችልም ፡፡ነገር ግን የትናቱ ተሳታፊ በመሆንዎ በአገልግሎት አሰጣጡ ችግሮችን በመለየት የተሻለ አገልግሎት እንድንሰጥ ያግዘናል ፡፡ስለሆነም የዚህ ጥናት መሳካት የርሰዎ አስተዋፅኦ ታላቅ ነዉ፡፡ በመሆኑም ለአሰተዋፅኦዎ በአፀፋዉ ታላቅ ምስጋና እለግሰዎታለሁ፡፡ሀሳቡን በትክክል ተረድተዉታል መጠይቁን መቀጠል እንችላለን?

1. ፈቃደኛ ነኝ ☐ ☐ 2. አይደለሁም

I:ክፍልአንድ፡- የማህበራዊ እና ስነ-ህዝባዊ ጥያቄዎች

ተ.ቁ	ጥያቄ	መልስ
101	ዕድሜ	በዓመት------------------
102	የመኖሪያቦታ	1. ገጠር 2. ከተማ
103	የጋብቻሁኔታ	1. ያገባች 2. የፈታች 3. የሞተባት<br>4. ያላገባች 5. የተለያዩ
104	ሀይማኖት	1. ኦርቶዶክስ 2. ሙስሊም<br>3. ፕሮቴስታንት 4. ካቶሊክ 5 ሌላ ካለ--------
105	ብሔር(ጎሳ)	1. አማራ 2. ኦሮሞ 3. አገው 4. ጉሙዝ 5. ሽናሻ 7 ሌላ (ይገለጥ) __________
106	ስራ	1. የቤት እመቤት 2. የመንግስት ሰራተኛ 3. የግል ሰራ<br>4. ገበሬ 5. ነጋዴ 6.

		የቀን ሰራተኛ<br>7. ተማሪ 8. ሌላ (ይገለጥ) ____________
107	የትምህርት ደረጃ	1. ማንበብ እና መፃፍ የማትችል 2. አንደኛ ደረጃ (1-8)<br>3. ሁለተኛደረጃ (9-12) 4. ኮሌጅ ወይም ዩኒቨርስቲ
108	የቤቱ አማካይ ወርሀዊ ገቢ ስንት ነዉ	የኢትዮጵያ ብር _________
109	የትዳር አጋርዎት የትምህርት ደረጃ	1. ማንበብ እና መፃፍ የማይችል 2. አንደኛ ደረጃ(1-8)<br>3. ሁለተኛ ደረጃ 4. ኮሌጅ ወይም ዩኒቨርስቲ
110	የትዳር አጋርዎት ስራ	1 ገበሬ 2. የመንግስትሰራተኛ 3. የግልሰራ<br>4. ነጋዴ 5. የቀንሰራተኛ 6. ሌላ (ይገለጥ) _____
111	የሚከተሉት የመገናኛ ዘዴዎች በቤትሽ ዉስጥ ይገኛሉ?	1. ሬድዮ 2. ቴሌቭዥን 3. የለም
112	በቅርብሽ ወደ ሚገኘዉ የጤና ተቋም በእግር ለመሄድ የሚፈጅብሽ ሰዓት ስንት ነዉ?	በሰዓት_________
ክፍልሁለት፡-ምቹ (አስቻይ)ሁኔታዎች እና የግንዛቤ ደረጃ		
201	የመጀመሪያ ልጅሽን በስንት አመትሽ ዎለድሽ?	በዓመት---------------
202	ስንት ልጆች በህዎት ዎለዱ	በቁጥር--------------
203	አጠቃላይ ስንት ጊዜ ዎልደዋል	በቁጥር---------------
204	በዎሊድ ጊዜ የልጅሽ ሂዎት ማለፍ አጋጥሞሽ ያቃል?	በቁጥር-----------------
205	የመጨረሻ እርግዝናሽ በእቅድ ነበር?	1. አዎ 2. አይደለም
206	የነፍሰጡር ክትትል ነበረሽ?	አዎ 2. የለም
207	ተ.ቁ 206 አዎ ከሆነ ሲንት ጊዜ?	በቁጥር---------------
208	የት ነው ክትተል ስታደርጊ የነበር?	1.ጤና ኬላ 2. ጤና ጣቢያ 3. ሆስፒታለል

209	ክትትል በምተሄጅበት ጊዜ ስለእርግዝናና ዎሊድ ላይ ስለሚከሰቱ ችግሮች መረጃ ሰምተሽል?	1.አዎ 2. የለም		
210	አዎ ከሆነ፣ ክትትል በምተሄጅበት ጊዜ የት መውለድ እንዳለብሽ መረጃ ሰጠውሻል?	1.ጠና ኬላ 2. ጤና ጣቢያ 3 ሆስፒታል		
211	በርግዝናሽ ላይ፣ከመውለድሽ በፊት ወይም በመውለድ ጊዜ የገጠመሽ ችግር ነበር?	1.አዎ 2. የለም		
212	ጥቁ 211፣አዎ፣ምን አይነት ችግር ነበር?	1. በወሊድ ጊዜ ደም መፍሰስ<br>2. በምጥ ጊዝ ከፍተኛ ደም መፍሰስ<br>3. የንሽርት ውሃ መፍሰስ<br>4. በማህፀን ውስጥ የፅንስ መሞት<br>5. ጊዜው ያልደረሰ ሞት<br>6. ከፍተኛ የደም ግፊት		
213	በጤና ተቋም መውለድ ጥቅሙ ምንድነው?		አዎ	አየለም
	ችግሮችን ቀድሞ ለመከላከል		1	2
	ለናትየዋ ለተሽለ የጤና አገልግሎት		1	2
	ለህጻኑ ለተሻለ የጤና አገልግሎት		1	2
214	በወሊድ ጊዜ ምን አየነት ችግር ሊከሰት ይችላል?		አዎ	አይደለም
		ከፍተኛ ደም መፍሰስ	1	2
		የአቀማመጥ ችግር	1	2
		ከ30 ደቂቃ በላይ የእንግዴ ልጅ አለመውረድ	1	2
		የማህጸን በር መጥበብ	1	2
		ከ 12 ሰአት በላይ የምጥ መራዘም	1	2
		የህጻን መታነቅ	1	2

ከፍልሶስት፡የጤና ተቋም አገልግሎት ሁኔታ

301	በቀበሌው ከ5 ኪሜ በታች ወይም በሁለት ሰዓት የግር ጉዞ ሊደረስበት የሚችል በሰለጠነ ባለሙያ የማዋለድ አገልግሎት የሚሰጥ ጤናተቋም አለ?	1. አዎ 2. የለም
302	ወደ ጤና ተቋም ለመሄድ መጓጓዣ ይገኛል?	1. ለአዎ 2. የለም
303	ለትራነስፖርት ክፍያ ለመክፈል በቂ አቀም አለሽ?	1. ለአዎ 2. የለም
	ስለጤና ተቋም የወሊድ አገልግሎት ምን	

	ታስቢያለሽ?	
304	ጤናማ የወሊድ አገልግሎት ለማግኘት ይጠቅማል	1.አዎ 2. የለም 3. አላቅም
305	የወሊድ ችግሮችን ለመከላከል	1. አዎ 2. የለም 3. አላቅም
306	የተፈጠሩ የወሊድ ችግሮችን ለማከም	1. አዎ 2. የለም 3. አላቅም
307	የጤና ችግሮችን ቅድሚያ ለመለየት	1. አዎ 2. የለም 3. አላቅም
308	የተሻለ ጤና አገልግሎት ለናትየዋ ለማግኘት	1. አዎ 2. የለም 3. አላቅም
309	የተሻለ ጤና አገልግሎት ለ ህጻኑ ለማግኘት	1. አዎ 2. የለም 3. አላቅም
310	በወሊድ ጊዜ ሊፈጠር የሚችለው ችግር ለህጻኑ እና ለናቲቱ አስችጋሪ ስለሚሆን	1. አዎ 2. የለም 3. አላቅም
311	በጤና ተቋም ለመውለድ ያለውን ሁኔታ አንቺ እንዴት ታይዋለሽ?	1. በጣም ቀላል<br>2. ብዙም አየከብድም<br>3. በጣም ከባድ
312	ባለሽ ልምድስለ ጤና አገልግሎት አሰጣጥ ያለሽ አመለካከት ምንድን ነው?	1. ጥሩ 2. መጥፎ 3. አላቅም
313	ጥ.ተ.ቁ፣ 311 መልሰ መጥፎ ከሆነ፣ምክኒያትሽ ምንድን ነው?	1. አገልግሎት አሰጣጡ ጥራት የገደለው ስለሆነ<br>2. በአገልግሎት አሰጣጥ ውጤቱ ስላላረካኝ<br>3. የጤና ባለሙያዎች ሁኔታ<br>4. ክፍያው ዉድ ስለሆነ<br>5. ሌላ----------------
314	ጥ.ተ.ቁ፣ 311 መልሰ ጥሩ ከሆነ፣ ምክኒያትሽ ምንድን ነው?	1. ያገልግሎት አሰጣጡ ጥራት ስላለው<br>2. የጤና ባለሙያዎች አገልግሎት አሰጣጥ ጥሩ ስለሆነ<br>3. አገልግሎቱን ካገኘሁ በኋላ ሁጤቱ ስላረካኝ<br>4. ክፍያው ስለማይበዛ
315	ቤት ስለመውለድ ያለሽ አመለካከት ምን ይመስላል	1. ጥሩ 2. መጥፎ 3 አላቅም
316	ጥ.ተ.ቁ፣ 314 መልሰ ጥሩ ከሆነ፣ለምንድን ነው?	1. ምቾት ስለሚሰማኝ<br>2. የበተሰብ እንክብካቤ ለማግኘት<br>3. ጤና ተቋም ላይ ጥሩ ገጠመኝ ስለሌለኝ<br>4. የጤና በለሙያወች ስለማይመቹኝ
ክፍልአራት፡- የዎሊደ አገልግሎት ለይ የማህበረስብ እና የግል ተነሳሽነትን በተመለከተ		

401	የመጨረሻዉ እርግዝናሽ አቅደሽበት ነው	1. አዎ 2. አይደለም
402	የመውለጃ ቦታሽን ሚመርጥልሽ ማነው	2. ራሴ 2. ባለቤቴ 3. ሁለታችን 4. እናቴ 5. ሌላ----
403	ባለቤትሽ የት ብትዎልጅ ይመርጠል	1. ቤት 2. ጤና ተቋም 3. አላቅም
404	ባለቤትሽን ማን ቢያዋልድሽ ድሰ ይለዋል	1. ጤና ባለሙያ፣ 2 ልምድ አዋላጅ 3.ቤተሰብ
405	ሌሎች የበተሰብ አባላቶችሽ የት ብትዎልጅ የመርጠሉ	1. ቤት 2. ጤናተቋም 3. አላቅም
406	ሌሎች የበተሰብ አባላቶችሽ ማን ቢያዋልድሽ ደስ ይላቸዋል	1. ጤናባለሙያ፣ 2 ልምድአዋላጅ 3.ቤተሰብ
407	ማህበረሰቡ የት መውለድን ያበረታታል	1. ቤት 2. ጤና ተቋም 3. አላቅም
408	ማህበረሰቡ ማን እንዲያዋልድሽ ያበረታታል?	1 ጤና ባለሙያ፣<br>2 ልምድአዋላጅ<br>3 ቤተሰብ
409	ለመጨረሻ ጊዜ ስትዎልጅ የት ዎለድሽ?	1.ቤት ውስጥ 2.ጤና ተቋም ውስጥ
410	ለጥያቄ 409 መልስዎ ቤት ውስጥ ከሆነ ምክኒያቱ ምን ነበር?	1. ቤት ውስጥ መውለድ የበለጠ ምቾት ስለሚሰጠኝ<br>2. ከዚህ በፊት በነበረኝ ልምድ ተነስቼ<br>3. ጤና ተቋም መዉለድ ስለሚያስጠላኝ<br>4. ከዚህ በፊት መጥፎ አጋጣሚ ስላለኝ<br>5. የጤና ባለሙያዎች ጥሩ ያልሆነ አቀባበል<br>6. ጤና ጰቋሙ ሩቅ ስለሆነ<br>7. ምጤ አጣዳፊ ስለነበር<br>8. መጓጓዣ ስለሌለ<br>9. በባለቤቴ ግፊት<br>10.በቤተሰቤ ፍላጎት<br>11.በሌሎች ምክኒያቶች------
411	ለጥያቄ 409 መልስዎ ጤና ተቋም ከሆነ ምክኒያቱ ምን ነበር?	1. ጥሩ አገልግሎት ለማገኘት<br>2. ለራሴ እና ለልጀ ምለካም ጤንነት<br>3. መጥፎ ገጠመኝ ስለነበረብኝ<br>4. ጤና ተቋም እንድዎልድ መረጃ ስለተሰጠን<br>5. ጤና ተቋሙ ቅርብ ስለሆነ<br>6. ሌሎች ምክኒያቶች------
412	ለጥያቄ 409 መልስዎ ጤና ተቋም ከሆነ ምን?	1. ጤና ኬላ

	አይነት ጤና ተቋም ውስጥ ወለዱ	2. ጤና ጣቢያ<br>3. ሆስፒታል

<u>አመሰግናለሁ</u>

የጠያቂው ስም ---------------------------

ቀን ---------------------------

ፊርማ -----------------------
