# Supplementary material for: Institutional delivery services utilization and associated factors among mothers who gave birth in the last year in Mandura district, Northwest Ethiopia
Source: PLoS One. 2020 Dec 16;15(12):e0243466. doi: 10.1371/journal.pone.0243466 (PMC7743934; doi:10.1371/journal.pone.0243466)
Supplement: S2 File — (PDF) [file pone.0243466.s002.pdf]

English version questionnaire

Institutional Delivery Service Utilization and associated Factors among Rural and Urban Mothers Who Gave Birth in Mandura district, Benshangul Gumuze, Western Ethiopia. 2019

INSTRUCTIONS

1. All questions are to be addressed to mothers who gave birth the last 1 year prior to data collection

Part I Socio Economic & demographic factors

S. NO	Questions	Answer to questions
101	Age	In years---------------
102	Residence	1. Rural<br>2. Urban
103	Marital status	1. Married<br>2. Divorced<br>3. Widowed<br>4. Single<br>5. Separated
104	Religion	1. Orthodox<br>2. Muslim<br>3. Protestant<br>4. Catholic<br>5. Others(specify)______
105	Ethnicity	1. Amhara<br>2. Oromo

		3. Agew<br><br>4. Gumez<br><br>5. Shenasha<br><br>6. Others, specify ________
106	Occupation	1. House wife<br><br>2. Governmental Employee<br><br>3. Private Employee<br><br>4. Farmer<br><br>5. Merchant<br><br>6. Daily Laborer<br><br>7. Student<br><br>8. Others, specify __________
107	Mother's educational Status	1. Unable to read and write<br><br>2. Primary education(1-8)<br><br>3. Secondary education(9-12)<br><br>4. College or University
108	Average monthly household in come	In Ethiopian Birr _____________
109	Husband's educational Status	1. Unable to read and write<br><br>2. Primary education(1-8)<br><br>3. Secondary and above (9-12)<br><br>4. College or University diploma and above

110	Husbands occupation	1. Farmer<br>2. Governmental employee<br>3. Private Employee<br>4. Merchant<br>5. Daily laborer<br>6. Other, specify____________
111	Do you have any of the following means of communication?	1. Radio<br>2. TV<br>3. None
112	How many hours do you take to reach the nearby health facility on foot (estimate by data collectors)?	In hour ______________

Part II Enabling factors (knowledge and obstetric questions)

201	What was your age at first birth	In years---------------
202	How many births have you ever had (parity)?	In numbers -----------------
203	Number of live births	In numbers -----------------
204	Number of still births if you ever had	In numbers -----------------
205	Did your last pregnancy planned?	1. Yes<br>2. No
206	Do you have ANC visit in the last pregnancy?	1. Yes<br>2. No
207	If Yes for **QN206**, how many times?	In numbers -----------------

208	Where did you attend ANC follow up?	1. Health Center<br>2. Hospital<br>3. Health post		
209	Did you get any information about pregnancy & delivery Complications during ANC follow up?	1. Yes<br>2. No		
210	If yes to **QN 209**, where did you recommended to deliver	1. Health facility 2. Home		
211	Did you face any problem during pregnancy or delivery?	1. Yes 2. No		
212	If yes to **QN 211** , which of the following problems you faced?	1. Ante partum hemorrhage<br>2. Excessive bleeding during labor<br>3. premature rupture of membranes<br>4. Intrauterine fetal death<br>5. preterm labor<br>6. Elevated blow pressure		
213	What are the advantages of attending delivery in health institutions?		Yes	No
		For anticipating problems	1	2
		For early detection of health Problems	1	2
		For better health care to the women	1	2
		For better care to the newborn	1	2
214	What are the complications that can occur during delivery?		Yes	No
		1. severe hemorrhage	1	2

		2. mal presentation and mal position	1	2
		3. retained placenta (lasting more than 30 minutes)	1	2
		4. Pelvic contracture	1	2
		5. prolonged labor (lasing more than 12 hours)	1	2
		6. Fetal distress	1	2
Part III:-Health facility and Health service factors				
301	Is there health facility with delivery attendant within 2 hours walking or within 5 km radius in the *kebele*	1. Yes 2. No		
302	Is there any transportation system to visit health facility (Can you got transportation services to visit Health Center with skill delivery attendant?)	1. Yes 2. No		
303	Can you afford to pay transportation services to visit Health Center?	1. Yes 2. No		
	What do you think about getting delivery service in health facility			
304	Attending Normal delivery	1. Yes 2. No 3. Do not know?		
305	Preventing delivery complications	1. Yes 2. No 3. Do not know?		
306	Managing delivery complications	1. Yes 2. No 3. Do not know?		
307	Early detection of Health Problems	1. Yes 2. No 3. Do not know?		
308	Better health care to the women	1. Yes 2. No 3. Do not know?		

309	Better care to the newborn	1. Yes 2. No 3. Do not know?
310	Delivery complications can be severe and may be hazardous to my well-being	1. Yes 2. No 3. Do not know?
311	Being attended by a skilled delivery attendant may be beneficial to my well-being	1. Yes 2. No 3. Do not know?
312	How did you rate the easiness for you to get institutional delivery services if the?	1. Very easy<br>2. It was impossible<br>3. Fair<br>4. I cannot assess.
IV. REINFORCING AND NEED FACTORS		
401	Was your last pregnancy planned?	1. Yes<br>2. No
402	Who was decision maker about the past place of delivery	1. Myself<br>2. My husband<br>3. Both of us<br>4. Mother- in- law<br>5. Others _____________
403	Preference of husband to place of delivery	1. Home Delivery 2. Institutional Delivery 3. Do not know
404	Preference of husband as your deliver attendant	2. Skilled Delivery attendant<br>3. Trained traditional Birth attendant<br>4. Traditional birth attendant<br>5. Relatives of family members<br>6. Do not know

405	Preference of other family members as your place of delivery	1. Home Delivery<br>2. Institutional Delivery<br>3. Do not know
406	Preference of other family members about your delivery attendant	1. Skilled Delivery attendant<br>2. Trained traditional Birth attendant<br>3. Traditional birth attendant<br>4. Relatives of family members<br>5. Do not know
407	Preference of other community members as your place of delivery	1. Home Delivery<br>2. Institutional Delivery<br>3. Do not know
408	preference of other community members as your delivery attendant	1. Skilled Delivery attendant<br>2. Trained traditional Birth attendant<br>3. Traditional birth attendant<br>4. Relatives of family members<br>5. Do not know
409	Where do you deliver your last child	1. Home 2. Health facility
410	If your answer to **QN 409** is **Home**, why did you prefer to deliver in home?	1. I feel more comfortable giving birth in home<br>2. Because it is my usual practice<br>3. I don't like the service in health facilities<br>4. I have bad experience in giving birth in health facilities<br>5. Unwelcoming approach of health workers in health facilities<br>6. the health facility is too far from my house

		7. labor was urgent to reach health facilities<br>8. lack of money for transport<br>9. influenced by my husband not go to health facilities<br>10. family members prefer to give birth in home<br>11. others reasons, specify--------
411	If your answer to **QN 409** is health facility, why did you choose to deliver in Health facility?	To get better services in health facilities<br>2. To get better outcomes from health facilities to me and my baby<br>3.Bad experience from past home delivery<br>4.I was informed to deliver in health facilities<br>5. The health facility closer to my home<br>6. Others, specify----------------
412	If you gave birth in health facilities , which health facility?	1. Health center<br>2.Hospital<br>3.Health post

Thank You

Interviewer Name ----------------------------

Date ---

Signature -------------------------------------
